# Global patterns of utilization of noninvasive tests for the clinical management of metabolic dysfunction–associated steatotic liver disease

**DOI:** 10.1097/HC9.0000000000000678

**Published:** 2025-04-30

**Authors:** Alina M. Allen, Jeffrey V. Lazarus, Naim Alkhouri, Mazen Noureddin, Vincent Wai-Sun Wong, Emmanuel A. Tsochatzis, Leyla de Avila, Andrei Racila, Fatema Nader, Henry E. Mark, Linda Henry, Maria Stepanova, Laurent Castera, Zobair M. Younossi

**Affiliations:** 1 The Global NASH Council, Washington, District of Columbia, USA; 2 Division of Gastroenterology and Hepatology, Mayo Clinic, Rochester, Minnesota, USA; 3 CUNY Graduate School of Public Health and Health Policy, New York, New York, USA; 4 Barcelona Institute for Global Health (ISGlobal), Barcelona, Spain; 5 Arizona Liver Health, Chandler, Arizona, USA; 6 Houston Methodist Hospital, Houston, Texas, USA; 7 Department of Medicine and Therapeutics, The Chinese University of Hong Kong, Sha Tin, Hong Kong; 8 UCL Institute for Liver and Digestive Health, Royal Free Hospital and UCL, London, UK; 9 Beatty Liver and Obesity Research Program, Inova Health System, Fairfax, Falls Church, Virginia, USA; 10 Department of Hepatology, Beaujon Hospital, Assistance Publique—Hôpitaux de Paris, Université Paris-Cité, Clichy, France; 11 Center for Outcomes Research in Liver Disease, Washington, District of Columbia, USA

**Keywords:** 2D-SWE, ELF, Fib-4, risk stratification, VCTE

## Abstract

**Background:**

Noninvasive tests (NITs) are used to risk-stratify metabolic dysfunction–associated steatotic liver disease. The aim was to survey global patterns of real-world use of NITs.

**Methods:**

A 38-item survey was designed by the Global NASH Council. Providers were asked about risks for advanced fibrosis, which NITs (cutoff values) they use to risk-stratify liver disease, monitor progression, and which professional guidelines they follow.

**Results:**

A total of 321 participants from 43 countries completed the survey (54% hepatologists, 28% gastroenterologists, and 18% other). Of the respondents, 85% would risk-stratify patients with type 2 diabetes, obesity (82%), or abnormal liver enzymes (73%). Among NITs to rule out significant or advanced fibrosis, transient elastography (TE) and fibrosis-4 (FIB-4) were most used, followed by NAFLD Fibrosis Score, Enhanced Liver Fibrosis, and magnetic resonance elastography. The cutoffs for ruling out significant fibrosis varied considerably between practices and from guidelines, with only 50% using TE <8 kPa, 65% using FIB-4 <1.30 for age <65, and 41% using FIB-4 <2.00 for age ≥65. Similar variability was found for ruling in advanced fibrosis, where thresholds of FIB-4 ≥2.67 and TE ≥10 kPa were used by 20% and 17%, respectively. To establish advanced fibrosis, 48% would use 2 NITs while 23% would consider 1 NIT, and 17% would confirm with liver biopsy. TE was used by >75% to monitor, and 66% would monitor (intermediate or high risk) annually. Finally, 65% follow professional guideline recommendations regarding NITs.

**Conclusions:**

In clinical practice, there is variability in NIT use and their thresholds. Additionally, there is suboptimal adherence to professional societies’ guidelines.

## INTRODUCTION

Metabolic dysfunction–associated steatotic liver disease (MASLD) is a public health challenge, with an estimated prevalence of 38% among adults worldwide.^1^^,^^2^ This condition is associated with substantial multisystemic implications, including an increased risk of major cardiovascular events, various malignancies, and end-stage liver disease.^3^^–^^5^ Recent data from the Global Burden of Disease study indicate that cirrhosis and other chronic liver diseases account for ~1.5 million deaths annually, and MASLD is growing in every region of the world.^6^^–^^8^ The World Health Organization identifies these diseases as the ninth leading cause of death in lower–middle-income countries.^9^ Notably, MASLD contributes to 9% of liver-related mortality and has seen a troubling increase in incidence, correlating with rising obesity and metabolic syndrome rates.^10^ In the United States, MASLD has become a predominant cause of end-stage liver disease necessitating transplantation, representing 30% of candidates with liver cancer and 26% of those without liver cancer as of 2022.^11^

Early identification of MASLD is critical for mitigating severe morbidity and mortality outcomes. The recent approval of resmetirom as the first therapeutic agent for metabolic dysfunction–associated steatohepatitis (MASH) underscores the urgent need for effective noninvasive diagnostic strategies to identify patients with moderate to advanced fibrosis^12^ Professional societies have provided care pathway recommendations for identifying individuals at high risk for advanced fibrosis, with the Fibrosis-4 (FIB-4) score recommended as a first-line noninvasive test (NIT), followed by secondary testing such as transient elastography (TE) and other NITs depending on availability.^13^^–^^17^ Despite these guidelines, real-world studies have revealed substantial gaps in adherence and variability in the use of NITs across different regions.^18^ Additionally, survey data indicate substantial heterogeneity in the NIT cutoffs used by clinicians to identify patients at risk for advanced liver disease.^19^ Limitations of these studies include inadequate assessment of NIT sequencing, decision-making processes based on cutoffs, small sample sizes, and gaps in real-world practice evaluation in various regions.

This study aims to address these gaps by investigating the availability and utilization of NITs, including the sequence of their use, decision-making cutoffs, and their application in distinguishing clinically significant from advanced fibrosis. Understanding these patterns is crucial, as a lack of consensus on optimal NIT usage and cutoffs may lead to variability in case detection, identification of candidates for therapy, and potential implications for patient outcomes and healthcare costs.^20^^,^^21^

## METHODS

The 38-item survey was developed by the members of The Global NASH Council. In the survey, participating healthcare providers, including physicians and Advanced Practice Providers, were asked about risk factors for advanced fibrosis in MASLD, which NITs they use in their clinical practice to exclude and to identify significant (F2–F4) and advanced (F3–F4) fibrosis, and how they prefer to monitor their MASLD patients.

The providers were first asked to provide their basic demographics (age, sex, country, and region of practice), clinical experience (professional degree, primary and secondary area of specialization, clinic setting, the number of years in practice), and the number of patients with MASLD/NAFLD or MASH/NASH they see per month (new or follow-up) and how those patients are identified at or referred to their practice.

The second part of the survey asked which risk factors the survey completers would consider when deciding to assess patients with known or suspected MASLD/NAFLD for advanced fibrosis; the multiple answer options included 17 different medical conditions (eg, Obesity) and an option to check “Everyone with suspected MASLD/NAFLD.”

In the third part of the survey, participants were asked which NITs for fibrosis they had available in their practice. The selection of NITs included FIB-4, NAFLD Fibrosis Score (NFS), FibroTest, Enhanced Liver Fibrosis (ELF), TE, FibroScan-AST, magnetic resonance elastography (MRE), 2D-shear wave elastography, Agile 3+, and Agile 4.

Then, the participants were asked to identify which NITs they use to *exclude (rule out)* patients at risk of clinically significant fibrosis (F2–F4) and, separately, advanced fibrosis (F3–F4) in their clinical practice. The NIT selection procedure included the order in which the selected NITs would be used (eg, the first choice NIT to exclude advanced F3–F4 fibrosis is FIB-4, the second choice NIT is ELF, the third choice is TE, etc.) and a list of cutoff values to choose from for each of the selected NITs (derived from literature, with a type-in option).

The providers were also asked how they used NITs to *identify (rule in)* patients at risk of advanced fibrosis (F3–F4) in their clinical practice. For this item, participants were requested to select the most common NITs they used, the respective cutoff values to rule in advanced fibrosis, and the corresponding action. The action options, if the first NIT cutoff was met, included making a definitive diagnosis of advanced fibrosis, referring to specialty care for assessment of the suspected diagnosis, confirming the suspected diagnosis with another NIT, or confirming it with a liver biopsy, or doing nothing despite the absence of a definitive diagnosis. Participants were also asked to choose their action in a given scenario when their first-line NIT was FIB-4 and it was in the indeterminate range (no specific range was provided; the options to choose from included ordering a biopsy, ordering another NIT, referring to a different specialist, or doing nothing), whether they would use different NITs or different cutoff values for patients with diabetes, and for which overall proportion of their MASLD/NAFLD patients outside of clinical trials they performed (or refer for) a liver biopsy.

The fourth section of the survey included questions about monitoring patients with MASLD. The subjects were asked if they monitored disease progression or response in their MASLD patients and, if yes, with which NIT and at what interval; these questions were asked separately for MASLD patients with low, intermediate, and high risk of advanced fibrosis (without specifying the criteria). Finally, the subjects were asked whether they used a formal/written national/professional society risk-stratification pathway and, if yes, to type in the name of the pathway.

The survey was self-administered through an online survey platform and was completed by participating healthcare providers using their own electronic devices. The online survey required a click to confirm participation that was displayed before any survey questions appeared so that subjects were able to read the informed consent first and then click “Yes” to agree and begin the survey; an option to click “No” and close the page was also provided. The informed consent stated that the respondents could refrain from answering any question that they preferred not to answer either by selecting the “Decline to answer” option or by skipping the question. The survey was strictly anonymous, and no personal identifiers were collected; this was a one-time questionnaire, and the subjects will not be recontacted.

The survey was to be completed in English. Each participating site disseminated the link to the survey to various specialists (Hepatology, Gastroenterology, and Medicine) in their home country between December 2023 and July 2024. All the answers were centrally stored in a secure electronic database and were analyzed in aggregate.

### Statistical analysis

The target sample size for this study was chosen to be 200. Only subjects who finished completing the survey and pressed the “Submit” button were included in the study sample. However, the electronic data collection system did not require answering all the questions in order to submit the survey; in case of missing data, only observed data was used for all analyses.

The distributions of answers have been summarized as N (%) or mean±SD and compared between groups of subjects using chi-square or Kruskal–Wallis nonparametric tests.

All analyses were conducted in SAS 9.4 (SAS Institute).

## RESULTS

### Participants characteristics

A total of 321 participants from 43 countries completed the survey (Table 1 and Supplemental Table S1, http://links.lww.com/HC9/B946). Of these, 54% reported their primary specialization being hepatology, 28% gastroenterology, and 18% another discipline; 77% also reported having a secondary specialization (Table 1). In addition, 77% had at least 10 years of practice, 85% worked in an academic or hospital setting, 90% were MD, 7% were advanced practice providers, and 72% saw at least 20 MASLD patients per month (Table 1).

**TABLE 1:** Demographic parameters of the survey completers

	Hepatologists	Gastroenterologists	Others	*p*	All
	173	91	57		321
What is your age group? n (%)					
25–34 years	16 (9.2)	9 (9.9)	7 (12.3)	0.80	32 (10.0)
35–44 years	42 (24.3)	34 (37.4)	19 (33.3)	0.07	95 (29.6)
45–54 years	54 (31.2)	22 (24.2)	13 (22.8)	0.31	89 (27.7)
55–70 years	49 (28.3)	25 (27.5)	15 (26.3)	0.96	89 (27.7)
Above 70 years	12 (6.9)	1 (1.1)	3 (5.3)	0.12	16 (5.0)
Male gender	112 (64.7)	59 (65.6)	23 (40.4)	0.0025	194 (60.6)
Primary specialization/discipline, n (%)					
Primary Care or General Medicine	0 (0.0)	0 (0.0)	19 (33.3)		19 (5.9)
Gastroenterology	0 (0.0)	91 (100.0)	0 (0.0)		91 (28.3)
Hepatology	173 (100.0)	0 (0.0)	0 (0.0)		173 (53.9)
Endocrinology	0 (0.0)	0 (0.0)	26 (45.6)		26 (8.1)
Medical weight loss	0 (0.0)	0 (0.0)	4 (7.0)		4 (1.2)
Other	0 (0.0)	0 (0.0)	8 (14.0)		8 (2.5)
Secondary specialization/discipline, n (%)					
Primary Care or General Medicine	5 (2.9)	7 (7.7)	5 (8.8)	0.11	17 (5.3)
Gastroenterology	107 (61.8)	7 (7.7)	0 (0.0)	<0.0001	114 (35.5)
Hepatology	15 (8.7)	57 (62.6)	9 (15.8)	<0.0001	81 (25.2)
Other including Endocrine and Medical Weight loss	13 (7.5)	2 (7.5)	21 (7.5)	<0.0001	23 (7.2)
None	33 (19.1)	18 (19.8)	22 (38.6)	0.0070	73 (22.7)
Number of years in practice? n (%)					
<5 years	10 (5.8)	14 (15.4)	8 (14.0)	0.0247	32 (10.0)
5–10 years	18 (10.4)	17 (18.7)	6 (10.5)	0.14	41 (12.8)
11–20 years	59 (34.1)	28 (30.8)	20 (35.1)	0.82	107 (33.3)
>20 years	86 (49.7)	32 (35.2)	23 (40.4)	0.06	141 (43.9)
Clinic setting, n (%)					
Academic	102 (59.0)	32 (35.2)	26 (45.6)	0.0009	160 (49.8)
Hospital	54 (31.2)	38 (41.8)	21 (36.8)	0.22	113 (35.2)
Private practice	15 (8.7)	21 (23.1)	8 (14.0)	0.0053	44 (13.7)
Other	2 (1.2)	0 (0.0)	2 (3.5)	0.17	4 (1.2)
Medical degree, n (%)					
MD	158 (91.3)	81 (89.0)	50 (87.7)	0.68	289 (90.0)
DO	1 (0.6)	1 (1.1)	0 (0.0)	0.71	2 (0.6)
NP or PA	12 (6.9)	7 (7.7)	2 (3.5)	0.58	21 (6.5)
Dietitian or Nutritionist	0 (0.0)	0 (0.0)	2 (3.5)	0.0095	2 (0.6)
Exercise Specialist	1 (0.6)	1 (1.1)	0 (0.0)	0.71	2 (0.6)
Other	1 (0.6)	1 (1.1)	3 (5.3)	0.0426	5 (1.6)
Region of practice, n (%)					
A large city or urban area	138 (79.8)	65 (71.4)	44 (77.2)	0.31	247 (76.9)
A medium-sized city or suburb	31 (17.9)	23 (25.3)	10 (17.5)	0.32	64 (19.9)
A small town or rural area	4 (2.3)	3 (3.3)	3 (5.3)	0.54	10 (3.1)
MASLD patients seen per month (new or follow-up), n (%)					
None	0 (0.0)	0 (0.0)	3 (5.3)	0.0009	3 (0.9)
1–19	37 (21.4)	30 (33.0)	21 (36.8)	0.0285	88 (27.4)
20–49	59 (34.1)	28 (30.8)	21 (36.8)	0.74	108 (33.6)
>50	77 (44.5)	33 (36.3)	12 (21.1)	0.0062	122 (38.0)
Patients with MASLD/NAFLD or MASH/NASH are referred from, n (%)					
Primary care	140 (80.9)	69 (75.8)	28 (49.1)	<0.0001	237 (73.8)
Other specialty such as Endocrinology	113 (65.3)	49 (53.8)	21 (36.8)	0.0006	183 (57.0)
Own practice	103 (59.5)	56 (61.5)	37 (64.9)	0.77	196 (61.1)

The distribution of the subjects by the country of practice is shown in Supplemental Table S1, http://links.lww.com/HC9/B946.

Abbreviations: DO, Doctor of Osteopathic Medical; MASH, metabolic dysfunction–associated steatohepatitis; MASLD, metabolic dysfunction–associated steatotic liver disease; MD, Medical Doctor; NP, Nurse Practitioner; PA, Physician's Assistant.

### Use of NITs for baseline risk stratification

**FIGURE 1:**
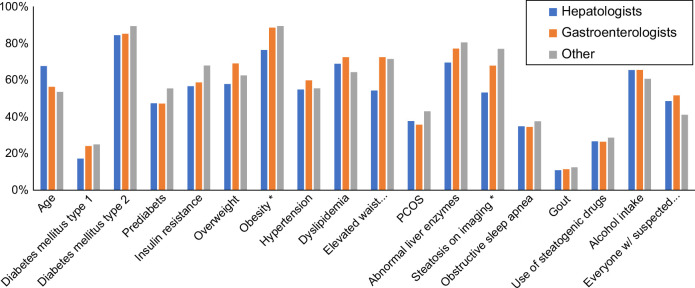
Distribution of risk factors selected by the survey participants (“Which risk factors do you consider when deciding to assess patients for advanced fibrosis in known or suspected MASLD/NAFLD (multiple choice)”). *p<0.05 across the 3 groups. Abbreviations: MASLD, metabolic dysfunction–associated steatotic liver disease; PCOS, Polycystic ovary syndrome.

Among the multidisciplinary respondents, 48% would use NITs to risk stratify all MASLD patients, while 85% would do so for known or suspected MASLD/NAFLD patients with type 2 diabetes, obesity (82%), or abnormal liver enzymes (73%). The reasons for risk stratification were distributed similarly across the medical specialization groups, with the exception of 3 circumstances in which hepatologists were less likely to risk stratify: obesity, elevated waist circumference, and steatosis on imaging (*p*<0.05) (Figure 1).

**FIGURE 2:**
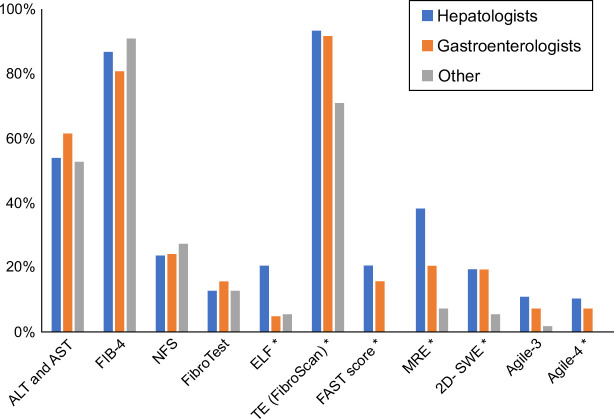
Availability of NITs for assessment of significant or advanced fibrosis in their clinical practice reported by the survey completers. Abbreviations: 2D-SWE, 2D-shear wave elastography; ELF, enhanced liver fibrosis; FAST score, FibroScan-AST score; FIB-4, fibrosis-4; MRE, magnetic resonance elastography; NFS, NAFLD Fibrosis Score; NITs, noninvasive tests; TE, transient elastography.

Among those who responded to the NIT-related questions (n=303), TE for assessment of fibrosis was available to 89% (93% hepatologists, 92% gastroenterologists, 71% others), FIB-4 to 86% (similar across the specialties), and MRE to 28% (38% hepatologists, 21% gastroenterologists, 7% others); other NITs (NFS, FibroTest, ELF, FibroScan-AST, 2D-shear wave elastography, Agile 3+, Agile-4) were available in their clinical practices to 8%–24% providers, more commonly to hepatologists (*p*<0.05) (Figure 2).

Among the NITs most used for ruling out significant or advanced fibrosis, TE (90%) and FIB-4 (76% for significant fibrosis, 65% for advanced fibrosis) were the most common options, followed by NFS, ELF, and MRE (chosen by 12%–17%; ELF and MRE were significantly more commonly chosen by hepatologists) (Table 2 and Supplemental Table S2, http://links.lww.com/HC9/B946). Furthermore, of the survey responders, 57% would use FIB-4 as their first choice NIT to exclude significant fibrosis (F2–F4) and 14% as their second choice, with the most commonly chosen FIB-4 cutoff being 1.30 for patients <65 years of age (chosen by 66% of FIB-4 users), 2.0 for patients ≥65 (41%), with no consistent differences between medical specialties (all but one *p*>0.05) (Table 2 and Supplemental Table S2, http://links.lww.com/HC9/B946). To exclude advanced fibrosis (F3–F4), 49% would still use FIB-4 as their first choice NIT and 13% as their second choice, although with a weaker consensus regarding cutoff values: the same most common options of 1.30 for <65 and 2.0 for ≥65 were chosen by only 42% and 27% of FIB-4 users, respectively, also similarly across the medical specialty groups (*p*>0.05) (Table 2 and Supplemental Table S2, http://links.lww.com/HC9/B946).

**TABLE 2:** The utilization of NITs to exclude (rule out) the risk of significant (F2–F4) or advanced (F3–F4) fibrosis in patients with MASLD/NAFLD

Question	Significant fibrosis (F2–F4)	Advanced fibrosis (F3–F4)
Select the most common NITs you use to exclude (rule out) patients at risk of fibrosis in your clinical practice, n (%)		
ALT and AST	55 (20.7)	45 (17.4)
*FIB-4*	*201 (75.6)*	*168 (64.9)*
NFS	32 (12.0)	30 (11.6)
FibroTest	11 (4.1)	10 (3.9)
ELF	34 (12.8)	30 (11.6)
*TE (FibroScan)*	*238 (89.5)*	*233 (90.0)*
FAST score	18 (6.8)	10 (3.9)
MRE	43 (16.2)	44 (17.0)
2D-SWE	21 (7.9)	20 (7.7)
Agile-3	5 (1.9)	11 (4.2)
Agile-4	3 (1.1)	5 (1.9)
The order of NIT use and respective NIT cutoffs, n (%)		
FIB-4 is the first NIT of choice	151 (56.8)	126 (48.6)
FIB-4 is the second NIT of choice	38 (14.3)	33 (12.7)
(<65 years) FIB-4 <1.00	6 (3.1)	2 (1.2)
(<65 years) FIB-4 <1.30	126 (65.6)	69 (42.1)
(<65 years) FIB-4 <1.45	33 (17.2)	24 (14.6)
(<65 years) FIB-4 <2.00	8 (4.2)	9 (5.5)
(<65 years) FIB-4 <2.67	14 (7.3)	39 (23.8)
(<65 years) FIB-4 <3.25	3 (1.6)	20 (12.2)
(<65 years) FIB-4 <other value	2 (1.0)	1 (0.6)
(≥65 years) FIB-4 <1.00	3 (1.6)	1 (0.6)
(≥65 years) FIB-4 < 1.30	38 (19.8)	19 (11.6)
(≥65 years) FIB-4 <1.45	43 (22.4)	35 (21.3)
(≥65 years) FIB-4 <2.00	79 (41.1)	45 (27.4)
(≥65 years) FIB-4 <2.67	22 (11.5)	30 (18.3)
(≥65 years) FIB-4 <3.25	6 (3.1)	32 (19.5)
(≥65 years) FIB-4 <other value	1 (0.5)	2 (1.2)
NFS is the first NIT of choice	8 (3.0)	6 (2.3)
NFS is the second NIT of choice	19 (7.1)	17 (6.6)
NFS <−1.455	23 (67.6)	14 (48.3)
NFS <0.675	11 (32.4)	15 (51.7)
ELF is the first NIT of choice	4 (1.5)	6 (2.3)
ELF is the second NIT of choice	15 (5.6)	12 (4.6)
ELF <7.7	13 (39.4)	4 (13.8)
ELF <9.8	19 (57.6)	20 (69.0)
ELF <11.3	1 (3.0)	5 (17.2)
TE is the first NIT of choice	80 (30.1)	103 (39.8)
TE is the second NIT of choice	126 (47.4)	106 (40.9)
TE <6 kPa	13 (5.6)	7 (3.1)
TE <7 kPa	54 (23.3)	22 (9.6)
TE <8 kPa	115 (49.6)	62 (27.2)
TE <9 kPa	23 (9.9)	32 (14.0)
TE <10 kPa	17 (7.3)	57 (25.0)
TE <11 kPa	2 (0.9)	6 (2.6)
TE <12 kPa	6 (2.6)	38 (16.7)
TE <other value	2 (0.9)	4 (1.8)
MRE is the first NIT of choice	6 (2.3)	5 (1.9)
MRE is the second NIT of choice	8 (3.0)	11 (4.2)
MRE <3.14 kPa	18 (41.9)	7 (15.9)
MRE <3.3 kPa	13 (30.2)	8 (18.2)
MRE <3.5 kPa	10 (23.3)	14 (31.8)
MRE <3.6 kPa	2 (4.7)	11 (25.0)
MRE <other value	0 (0.0)	4 (9.1)

The distributions of answers by the medical specialty are shown in Supplemental Table S2, http://links.lww.com/HC9/B946.

Abbreviations: 2D-SWE, 2D-shear wave elastography; ELF, Enhanced Liver Function; FAST, FibroScan-AST; FIB-4, fibrosis-4; MASLD, metabolic dysfunction–associated steatotic liver disease; MRE, magnetic resonance elastography; NFS, NAFLD Fibrosis Score; NIT, noninvasive test; TE, transient elastography.

TE was identified as the first choice NIT to exclude significant fibrosis (F2–F4) by 30% of the survey completers (although only by 11% of nonspecialists), and by 47% as their second choice, with the most common cutoff value being 8 kPa (50%) followed by 7 kPa (23%) (Table 2 and Supplemental Table S2, http://links.lww.com/HC9/B946). To exclude advanced fibrosis (F3–F4), TE was the first choice for 40% and the second choice for 41%, similar across specialties (*p*>0.05), with the most common cutoffs being 8 kPa (27%) and 10 kPa (25%), and hepatologists’ and gastroenterologists’ choices skewed toward higher cutoff values (12 kPa was chosen by 16% hepatologists, 25% gastroenterologists, and 3% of other providers, *p*=0.027) (Table 2 and Supplemental Table S2, http://links.lww.com/HC9/B946). The next most commonly used NITs (NFS, ELF, and MRE) were the first or second choices for excluding significant or advanced fibrosis in MASLD for 5%–10% of provider responders (Table 2).

For identifying (ruling in) advanced fibrosis in MASLD, the most commonly used rules were FIB-4 ≥2.67 (20%), TE ≥10 kPa (17%), TE ≥8 kPa (11%), or TE ≥12 kPa (11%), the latter chosen more commonly by hepatologists (14%, *p*=0.03) (Table 3). To definitively diagnose advanced fibrosis, 48% of respondents would use at least 2 NITs, while 23% would consider 1 NIT as sufficient (9% of nonspecialists), and 17% would confirm with a liver biopsy, similarly across the medical specialties (*p*>0.05) (Table 3). Only 10% would use a different cutoff than the standard cutoff for patients with type 2 diabetes (Table 3). Overall, the majority of the providers (64%), regardless of the medical specialty, would rarely (<10%) refer their MASLD/NAFLD patients for a biopsy outside of clinical trials (Table 3).

**TABLE 3:** The use of NITs to identify MASLD/NAFLD patients at risk of advanced (F3–F4) fibrosis

Question	Hepatologists	Gastroenterologists	Others	*p*	All
Do you use NITs to identify patients at risk of advanced fibrosis (F3–F4) in your clinical practice? N (%) “Yes”	149 (90.3)	72 (84.7)	45 (81.8)	0.19	266 (87.2)
Select the most common first-line NIT you use to identify patients at risk for advanced fibrosis and the cutoff value:					
FIB-4 >1.30	7 (4.7)	7 (10.0)	7 (15.9)	0.0420	21 (8.0)
FIB-4 >1.45	7 (4.7)	6 (8.6)	2 (4.5)	0.48	15 (5.7)
FIB-4 >2.67	33 (22.1)	9 (12.9)	11 (25.0)	0.19	53 (20.2)
FIB-4 >3.25	11 (7.4)	6 (8.6)	5 (11.4)	0.70	22 (8.4)
TE >8 kPa	15 (10.1)	7 (10.0)	8 (18.2)	0.30	30 (11.4)
TE >9 kPa	11 (7.4)	3 (4.3)	0 (0.0)	0.14	14 (5.3)
TE >10 kPa	24 (16.1)	16 (22.9)	5 (11.4)	0.25	45 (17.1)
TE >2 kPa	21 (14.1)	8 (11.4)	0 (0.0)	0.0318	29 (11.0)
TE >15 kPa	8 (5.4)	2 (2.9)	0 (0.0)	0.23	10 (3.8)
MRE >3.6 kPa	4 (2.7)	1 (1.4)	1 (2.3)	0.84	6 (2.3)
Other NIT/cutoff	8 (5.4)	5 (7.1)	5 (11.4)	0.38	18 (6.8)
Second step after the first condition is met:					
Confirm with another NIT	78 (53.1)	31 (44.3)	16 (36.4)	0.12	125 (47.9)
Do a biopsy	22 (15.0)	15 (21.4)	7 (15.9)	0.49	44 (16.9)
Refer to specialty care	4 (2.7)	2 (2.9)	16 (36.4)	<0.0001	22 (8.4)
Do nothing	5 (3.4)	5 (7.1)	1 (2.3)	0.34	11 (4.2)
The first step was sufficient to make a definitive diagnosis	38 (25.9)	17 (24.3)	4 (9.1)	0.06	59 (22.6)
If your first-line NIT is FIB-4 and it is in the indeterminate range, you (single choice):					
Perform a liver biopsy	26 (18.3)	12 (18.2)	2 (5.1)	0.12	40 (16.2)
Perform another NIT	110 (77.5)	45 (68.2)	19 (48.7)	0.0021	174 (70.4)
Refer to a specialist (GI/HEP)	2 (1.4)	6 (9.1)	17 (43.6)	<0.0001	25 (10.1)
Nothing	4 (2.8)	3 (4.5)	0 (0.0)	0.40	7 (2.8)
Do not know	0 (0.0)	0 (0.0)	1 (2.6)	0.07	1 (0.4)
Do you use different NITs or different cutoffs for people with diabetes? N (%) “Yes”	16 (11.3)	7 (10.3)	2 (5.4)	0.57	25 (10.1)
What proportion of your MASLD/NAFLD patients do you biopsy or refer for liver biopsy outside of clinical trials?					
0%–10%	85 (59.4)	46 (67.6)	28 (71.8)	0.26	159 (63.6)
11%–20%	29 (20.3)	15 (22.1)	6 (15.4)	0.70	50 (20.0)
21%–30%	18 (12.6)	4 (5.9)	0 (0.0)	0.0296	22 (8.8)
31%–40%	2 (1.4)	2 (2.9)	2 (5.1)	0.38	6 (2.4)
>40%	9 (6.3)	1 (1.5)	3 (7.7)	0.25	13 (5.2)

Abbreviations: FIB-4, fibrosis-4; GI, gastroenterologists; HEP, hepatologists; MASLD, metabolic dysfunction–associated steatotic liver disease; MRE, magnetic resonance elastography; NIT, noninvasive test; TE, transient elastography.

### Use of NITs for disease monitoring

**FIGURE 3:**
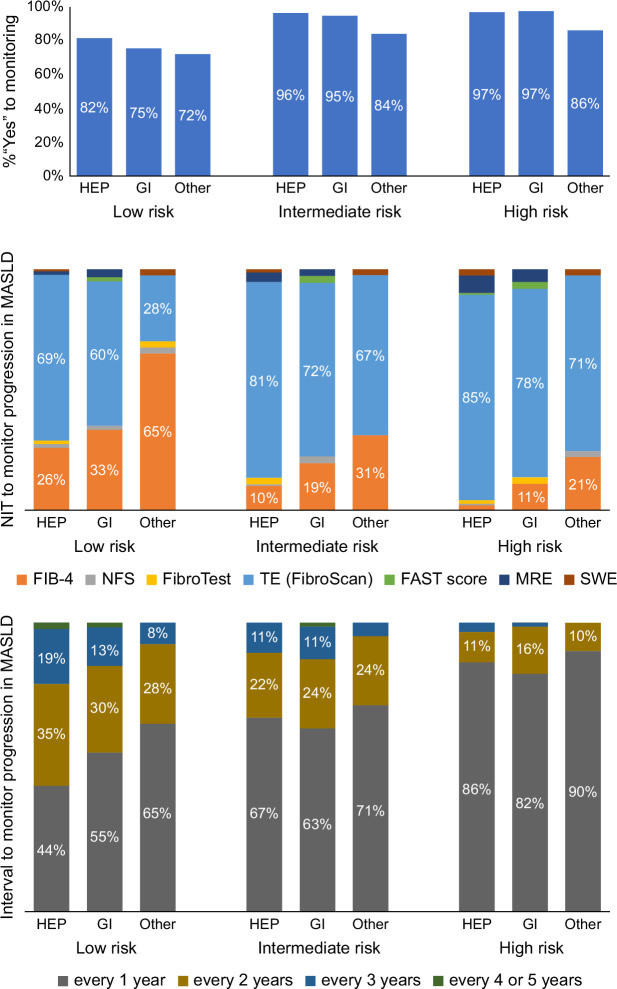
The choice of NITs and intervals for monitoring disease progression or response in MASLD/NAFLD patients with low, intermediate, or advanced risk of advanced fibrosis (“Do you monitor disease progression or response in MASLD/NAFLD patients with <low; intermediate; high> risk of advanced fibrosis? <if “Yes”> Which test? At what interval?”). Abbreviations: FAST, FibroScan-AST; FIB-4, fibrosis-4; GI, gastroenterologists; HEP, hepatologists; MASLD, metabolic dysfunction–associated steatotic liver disease; MRE, magnetic resonance elastography; NFS, NAFLD Fibrosis Score; NIT, noninvasive test; 2D-SWE, shear wave elastography; TE, transient elastography.

Most of the survey responders reported that they would monitor disease progression in all of their MASLD patients, including those at low risk of advanced fibrosis (overall “Yes” 78%), and 94% would do so for patients at an intermediate or advanced risk; the latter included 84% of nonspecialists (Figure 3). The most commonly used method for monitoring the disease progression in MASLD was TE (>60%), although nonspecialists would prefer to use FIB-4 for low-risk patients (65%) (Figure 3). Most providers (>63%) would monitor patients at intermediate or high risk every year (Figure 3).

### Use of professional society guidelines

Regarding the sources of risk-stratification care pathways used by the survey completers, 65% reported following at least one of such pathways provided by a national/professional society, with the most commonly followed ones being those by the American Association for the Study of Liver Diseases (AASLD) (43% including 8% nonspecialists) and European Association for the Study of the Liver (EASL) (22% including 38% nonspecialists) (Supplemental Table S3, http://links.lww.com/HC9/B946).

### Regional differences in practice patterns

Across regions of the world, Europe, North America, and Middle East/Africa each contributed 26% of survey completers, 17% were from Asia (South or Southeast), and 6% from Central and South America (Supplemental Table S4, http://links.lww.com/HC9/B946). Comparing the regions, hepatologists were overrepresented in Asia and North America (66% vs. 54% overall), providers from North America more commonly worked in a private practice setting (24% vs. 14% overall) and more commonly were advanced practice providers (eg, nurse practitioner or physician assistant) (21% vs. 7% overall) rather than medical doctors, while providers from Asia reported seeing more MASLD/NAFLD patients per month (60% saw >50 patients) (Supplemental Table S4, http://links.lww.com/HC9/B946). Regarding the availability of various NITs for fibrosis, the ELF and MRE were substantially more commonly available to providers from North America (36% vs. 14% overall and 56% vs. 28% overall, respectively), while >30% of providers from Latin America and Asia reported having access to 2D-shear wave elastography (17% overall) (Supplemental Table S4, http://links.lww.com/HC9/B946). However, the distributions of specific NIT-based rules for excluding, identifying, and monitoring patients with significant and/or advanced fibrosis were not consistently different between providers from different regions of the world (data not shown). Finally, there was a substantial geographic variability in the preference for risk-stratification care pathways recommended by national professional societies. Recommendations by AASLD were followed by 74% of providers from North America and 80% from Central/South America versus 7% from Europe who rather relied on that provided by EASL (41%) and 35% from Asia who predominantly chose local/regional professional societies (Indian, Korean, Taiwanese, etc.) (Supplemental Table S4, http://links.lww.com/HC9/B946).

## DISCUSSION

This comprehensive global survey of multidisciplinary healthcare practitioners revealed notable variability in the utilization of NITs for risk stratification in MASLD. We observed some heterogeneity in the types of NITs employed, their sequence of use, and the cutoff values used across different clinical settings. Moreover, longitudinal monitoring predominantly involves TE annually, even among individuals deemed at low risk. Practice guidelines from professional societies are adhered to by only 65% of practitioners, while the remainder rely on local protocols. Liver biopsy continues to be utilized outside of clinical trials, in higher proportions in regions such as Asia, Africa, and the Middle East.

Guidelines universally recommend the FIB-4 score as the first-line NIT for fibrosis risk stratification in individuals with metabolic risk factors (Table 4). The commonly endorsed cutoff values to rule out advanced fibrosis are 1.3 for individuals under 65 years and 2 for those 65 years and older. These cutoffs were selected based on high negative predictive values of 93% and high performance for the detection of advanced fibrosis (AUROC=0.79).^22^^,^^23^ The recommended cutoff for TE is 8 kPa, which can diagnose significant fibrosis with AUROC=0.77. An algorithm using these 2 NITs at these selected cutoffs has an negative predictive value of 90% to exclude advanced disease in the general US population.^24^ In this survey, only 57% of respondents used FIB-4 as the initial NIT for ruling out significant fibrosis and 49% for advanced fibrosis. In contrast, TE was used by 30% and 40% of respondents as the first-line test for ruling out significant and advanced fibrosis, respectively. This higher use of elastography likely reflects a specialty-driven practice pattern, as gastroenterologists and hepatologists comprised the majority of participants. In other surveys including a larger number of providers, elastography was used by a substantially lower proportion of primary care respondents when compared to hepatology specialists (10% vs. 65%).^25^ Nevertheless, even among specialists, there was considerable variability in the cutoffs applied for both FIB-4 and TE. For FIB-4, only 66% of participants used the recommended cutoff of 1.3 for individuals under 65 years to exclude moderate fibrosis, and 41% used the cutoff of 2 for those 65 years and older. These findings align with a previous survey indicating a range of cutoffs between 1 and 1.45 and highlight the need for standardization.^19^

**TABLE 4:** Summary of recommended NITs and cutoffs from the latest AASLD and EASL/EASO/EASD guidelines

	AASLD	EASL/EASD/EASO
First-line NIT	FIB-4	FIB-4
<1.3	Reassess in 2–3 years (1–2 y if DM)	Reassess in 1–3 years
1.3–2.67	Second-line NIT	Second-line NIT in individuals with T2DM OR Intensify management of comorbidities
Second-line NIT	VCTE, ELF	VCTE (or MRE, SWE, ELF with adapted thresholds)
VCTE <8 kPa, ELF <7.7	Reassess FIB-4	Reassess FIB-4
VCTE ≥ 8kPa, ELF ≥7.7	Hepatology referral	Hepatology referral
≥2.67	Hepatology referral	Hepatology referral

Abbreviations: AASLD, American Association for the Study of Liver Diseases; DM, diabetes mellitus; EASD, European Association for the Study of Diabetes; EASL, European Association for the Study of the Liver; EASO, European Association for the Study of Obesity; ELF, enhanced liver fibrosis; FIB-4, fibrosis-4; MRE, magnetic resonance elastography; NITs, noninvasive tests; SWE, shear wave elastography; T2DM, type 2 diabetes mellitus; VCTE, vibration controlled transient elastography.

The use of TE as a first-line NIT was reported by 30% of respondents for ruling out significant fibrosis and by 40% for advanced fibrosis. However, variability in TE cutoffs was also evident, with only 50% adhering to the guideline-recommended 8 kPa to identify patients at high risk for significant fibrosis. The range of cutoffs for identifying advanced fibrosis varied between 8 and 12 kPa. Other NITs, such as the ELF (4%–5%) and MRE (17%), were less commonly used globally, with a higher proportion in the United States.

Another key finding in this study underscores the importance of appropriate monitoring intervals for MASLD. Given the slow progression of the disease, during which liver outcomes affect a small proportion of people over decades and fibrosis advances ~1 stage every 7–14 years when assessed by histology, the frequency and type of monitoring should be tailored to the disease stage.^26^^,^^27^ These findings have been supported by studies correlating the severity of liver stiffness measured by MRE or TE with the probability of liver outcomes. Robust data support that early-stage disease can be monitored with FIB-4 every 2–3 years if the metabolic risk factors persist, as the probability of developing cirrhosis in this timeframe was <1%.^21^^,^^28^^–^^33^ Most guidelines do not make formal recommendations regarding the frequency of longitudinal monitoring in those with moderate to advanced fibrosis, which may allow heterogeneity in clinical practice. As the probability of cirrhosis development in those with moderate-advanced fibrosis is higher than in those with early disease, more frequent monitoring with elastography is reasonable, although an optimal frequency needs to be determined, and yearly would probably be warranted only in those with advanced fibrosis. The prevalent use of TE for annual monitoring revealed in this study, irrespective of disease stage, was excessive and may lead to unnecessary healthcare expenditures over decades. In those at low risk of fibrosis, only 35% were monitored with an accessible test such as FIB-4.

In this predominantly specialty-based cohort, confirmation of advanced fibrosis using a single NIT was reported by 23% of respondents, although the survey was not designed to explore the type and cutoff where a single NIT is insufficient. When a secondary diagnostic step was utilized, liver biopsy emerged as the second most frequently employed method, used by 17% of participants, while 48% would confirm with a second NIT. Furthermore, guidelines recommend utilizing an additional NIT when FIB-4 results are indeterminate,^13^^–^^17^ and this approach was adopted by 70% of respondents, whereas 16% would proceed directly to liver biopsy. The high usage of liver biopsy, particularly in Asia and the Africa/Middle East regions, where it was utilized by up to 35% and 26% of respondents, respectively, may be partly attributable to the limited availability of NITs, as indicated by 76% of responses from these regions. However, the relatively high use of liver biopsy in Asia, despite the predominant use of TE as the first-line NIT and lower FIB-4 utilization (34%), suggests additional factors at play. Regional disparities were further highlighted by the higher availability of MRE in the United States (56% of respondents) as compared to Europe (17%), and its use for ruling out advanced fibrosis in 17% of cases. Conversely, the ELF test, although most available in the United States (36%), was infrequently used (2% as first-line and 4%–5% as second-line testing). These findings underscore the need for greater consistency in NIT utilization and availability across different regions. Nevertheless, more research is needed to better understand structural incentives and disincentives in each country, including their reimbursement policies supporting these NITs.

Despite the recent publication of guidelines by multiple professional societies, real-world practice demonstrates significant heterogeneity and suboptimal adherence. Factors contributing to this disparity include the lack of consensus between guidelines on optimal cutoffs resulting in the heterogeneity in values used, as well as potential knowledge gaps. In a previous qualitative survey of 29 practitioners from multiple disciplines (primary care, diabetology, and hepatology) in Europe and the United States, the main barriers to optimal use of NITs were related to low awareness, suboptimal knowledge of NITs and the skills needed to interpret the values.^34^ Similar findings have been echoed in other studies, which suggest clinicians are uncertain how to interpret abnormal liver tests, what NITs are available and how to use them, which NITs are the most appropriate to detect clinically significant findings, and when to refer patients to specialists.^35^^–^^37^ Moreover, liver disease was not perceived as priority in primary care and there was reluctance to undertake specialty investigations since most primary care physicians feel that they do not have access to relevant, focused education in liver disease.^38^ As expressed by patients and providers, both groups would benefit from unified, evidence-based guidelines and educational initiatives to improve the consistency of NIT use.^39^

The recent conditional approval of the first therapeutic agent for MASH in non-cirrhotic patients with moderate to advanced fibrosis underscores the importance of identifying those who may benefit from this treatment. Given that the drug’s indication excludes individuals with early-stage MASH or those with cirrhosis, and that a liver biopsy is not mandated, the consistent application of appropriate NITs supported by robust evidence is crucial for both patient identification and longitudinal monitoring to assess therapeutic response. Analysis of the NIT values from 888 participants with stages 2–3 fibrosis in the phase 3 trial of resmetirom versus placebo (MAESTRO-NASH) has led to the proposal of benchmark cutoff values for different NITs by an expert panel.^40^ Notably, the median FIB-4 value in this cohort was 1.3 (IQR: 1.0–1.8), which is lower than anticipated given the histologic stages, thereby raising concerns about the utility of FIB-4 as a primary NIT for patient referral from primary care for treatment selection. Treatment decisions should thus rely on additional NITs, ideally incorporating more than one. In the MAESTRO-NASH cohort, the median liver stiffness measurement by VCTE was 10 kPa, so this cutoff was selected as the treatment selection benchmark.^12^ The discrepancy between this cutoff and the use of an 8 kPa cutoff in the other guidelines could potentially cause confusion by creating a new “gray zone” of 8–10 kPa, where only lifestyle interventions are currently recommended. These cutoffs may require reassessment as more data become available to streamline clinical decision-making pathways.

This study’s strengths include its global scope and detailed examination of MASLD care pathways, highlighting gaps in current practice and identifying areas for improvement. Limitations include the underrepresentation of primary care and non-hepatology practitioners, limited responses from certain regions such as North Africa, and insufficient data related to cutoffs for less commonly used NITs. Additionally, as in similar surveys, it would be impossible to cover all the nuances of NIT use. For example, specific high-risk groups, such as older patients, have not been explored.^41^

In summary, this in-depth survey of MASLD-related NITs from real-world practices suggests significant heterogeneity and deviation from recent guidelines. In this context, while NITs are widely used, there are substantial differences in availability, cutoff values, and practice patterns across the world. These discrepancies have implications for patient selection, monitoring decisions, and resource utilization. There is an urgent need for standardized, evidence-based risk-stratification pathways and enhanced educational efforts to align practices globally. Additionally, ongoing investment in developing and deploying accurate, affordable, and accessible NITs is essential.

## Supplementary Material

## References

[1] Younossi ZM Golabi P Paik JM Henry A Van Dongen C Henry L. The global epidemiology of nonalcoholic fatty liver disease (NAFLD) and nonalcoholic steatohepatitis (NASH): A systematic review. Hepatology. 2023;77:1335–1347. 36626630 10.1097/HEP.0000000000000004 PMC10026948

[2] Lazarus JV Mark HE Anstee QM Arab JP Batterham RL Castera L. NAFLD Consensus Consortium. Advancing the global public health agenda for NAFLD: A consensus statement. Nat Rev Gastroenterol Hepatol. 2022;19:60–78. 34707258 10.1038/s41575-021-00523-4

[3] Lee HH Lee HA Kim EJ Kim HY Kim HC Ahn SH. Metabolic dysfunction–associated steatotic liver disease and risk of cardiovascular disease. Gut. 2024;73:533–540. 37907259 10.1136/gutjnl-2023-331003

[4] Roderburg C Kostev K Mertens A Luedde T Loosen SH. Non-alcoholic fatty liver disease (NAFLD) is associated with an increased incidence of extrahepatic cancer. Gut. 2023;72:2383–2384. 36347594 10.1136/gutjnl-2022-328887

[5] Sanyal AJ Van Natta ML Clark J Neuschwander-Tetri BA Diehl A Dasarathy S. NASH Clinical Research Network (CRN). Prospective study of outcomes in adults with nonalcoholic fatty liver disease. N Engl J Med. 2021;385:1559–1569. 34670043 10.1056/NEJMoa2029349 PMC8881985

[6] Wu XN Xue F Zhang N Zhang W Hou JJ Lv Y. Global burden of liver cirrhosis and other chronic liver diseases caused by specific etiologies from 1990 to 2019. BMC Public Health. 2024;24:363. 38310221 10.1186/s12889-024-17948-6 PMC10837876

[7] Paik JM Henry L Younossi Y Ong J Alqahtani S Younossi ZM. The burden of nonalcoholic fatty liver disease (NAFLD) is rapidly growing in every region of the world from 1990 to 2019. Hepatol Commun. 2023;7:e0251. 37782469 10.1097/HC9.0000000000000251 PMC10545420

[8] Paik JM Golabi P Younossi Y Mishra A Younossi ZM. Changes in the Global Burden of Chronic Liver Diseases from 2012 to 2017: The growing impact of NAFLD. Hepatology. 2020;72:1605–1616. 32043613 10.1002/hep.31173

[9] WHO. The top 10 causes of death. Accessed September 4, 2024. https://www.who.int/news-room/fact-sheets/detail/the-top-10-causes-of-death.

[10] Golabi P Owrangi S Younossi ZM. Global perspective on nonalcoholic fatty liver disease and nonalcoholic steatohepatitis—Prevalence, clinical impact, economic implications and management strategies. Aliment Pharmacol Ther. 2024;59(suppl 1):S1–S9. 38813821 10.1111/apt.17833

[11] Younossi ZM Stepanova M Al Shabeeb R Eberly KE Shah D Nguyen V. The changing epidemiology of adult liver transplantation in the United States in 2013–2022: The dominance of metabolic dysfunction–associated steatotic liver disease and alcohol-associated liver disease. Hepatol Commun. 2023;8:e0352. 38126928 10.1097/HC9.0000000000000352 PMC10749707

[12] Harrison SA Bedossa P Guy CD Schattenberg JM Loomba R Taub R. MAESTRO-NASH Investigators. A phase 3, randomized, controlled trial of resmetirom in NASH with liver fibrosis. N Engl J Med. 2024;390:497–509. 38324483 10.1056/NEJMoa2309000

[13] Rinella ME Neuschwander-Tetri BA Siddiqui MS Abdelmalek MF Caldwell S Barb D. AASLD Practice Guidance on the clinical assessment and management of nonalcoholic fatty liver disease. Hepatology. 2023;77:1797–1835. 36727674 10.1097/HEP.0000000000000323 PMC10735173

[14] Wattacheril JJ Abdelmalek MF Lim JK Sanyal AJ. AGA Clinical Practice Update on the role of noninvasive biomarkers in the evaluation and management of nonalcoholic fatty liver disease: Expert review. Gastroenterology. 2023;165:1080–1088. 37542503 10.1053/j.gastro.2023.06.013

[15] European Association for the Study of the Liver (EASL); European Association for the Study of Diabetes (EASD); European Association for the Study of Obesity (EASO). EASL–EASD–EASO Clinical Practice Guidelines on the management of metabolic dysfunction–associated steatotic liver disease (MASLD). J Hepatol. 2024;81:492–542. 38851997 10.1016/j.jhep.2024.04.031

[16] Eslam M Sarin SK Wong VW Fan JG Kawaguchi T Ahn SH. The Asian Pacific Association for the Study of the Liver clinical practice guidelines for the diagnosis and management of metabolic associated fatty liver disease. Hepatol Int. 2020;14:889–919. 33006093 10.1007/s12072-020-10094-2

[17] Duseja A Singh SP De A Madan K Rao PN Shukla A. Indian National Association for Study of the Liver (INASL) Guidance Paper on nomenclature, diagnosis and treatment of nonalcoholic fatty liver disease (NAFLD). J Clin Exp Hepatol. 2023;13:273–302. 36950481 10.1016/j.jceh.2022.11.014 PMC10025685

[18] Anstee QM Hallsworth K Lynch N Hauvespre A Mansour E Kozma S. Real-world management of non-alcoholic steatohepatitis differs from clinical practice guideline recommendations and across regions. JHEP Rep. 2021;4:100411. 34977520 10.1016/j.jhepr.2021.100411 PMC8686034

[19] Lazarus JV Castera L Mark HE Allen AM Adams LA Anstee QM. Real-world evidence on non-invasive tests and associated cut-offs used to assess fibrosis in routine clinical practice. JHEP Rep. 2022;5:100596. 36644239 10.1016/j.jhepr.2022.100596 PMC9832273

[20] Younossi ZM Paik JM Henry L Yang J Fernandes G Stepanova M. The growing economic and clinical burden of nonalcoholic steatohepatitis (NASH) in the United States. J Clin Exp Hepatol. 2023;13:454–467. 37250870 10.1016/j.jceh.2022.12.005 PMC10213853

[21] Allen AM Lazarus JV Younossi ZM. Healthcare and socioeconomic costs of NAFLD: A global framework to navigate the uncertainties. J Hepatol. 2023;79:209–217. 36740046 10.1016/j.jhep.2023.01.026 PMC10293095

[22] Graupera I Thiele M Serra-Burriel M Caballeria L Roulot D Wong GL. Low accuracy of FIB-4 and NAFLD fibrosis scores for screening for liver fibrosis in the population. Clin Gastroenterol Hepatol. 2022;20:2567–2576.e6. 34971806 10.1016/j.cgh.2021.12.034

[23] Vali Y Lee J Boursier J Petta S Wonders K Tiniakos D. Biomarkers for staging fibrosis and non-alcoholic steatohepatitis in non-alcoholic fatty liver disease (the LITMUS project): A comparative diagnostic accuracy study. Lancet Gastroenterol Hepatol. 2023;8:714–725. 36958367 10.1016/S2468-1253(23)00017-1

[24] Udompap P Therneau TM Canning RE Benson JT Allen AM. Performance of American Gastroenterological Association Clinical Care Pathway for the risk stratification of patients with nonalcoholic fatty liver disease in the US population. Hepatology. 2023;77:931–941. 35989502 10.1002/hep.32739

[25] Porayko MK Articolo A Cerenzia W Coleman B Patel D Stacy S. Differences in NAFLD/NASH management by provider specialty: Opportunities for optimizing multidisciplinary care. J Multidiscip Healthc. 2022;15:1533–1545. 35898947 10.2147/JMDH.S367607 PMC9309172

[26] Allen AM Therneau TM Ahmed OT Gidener T Mara KC Larson JJ. Clinical course of non-alcoholic fatty liver disease and the implications for clinical trial design. J Hepatol. 2022;77:1237–1245. 35843374 10.1016/j.jhep.2022.07.004 PMC9974107

[27] Singh S Allen AM Wang Z Prokop LJ Murad MH Loomba R. Fibrosis progression in nonalcoholic fatty liver vs nonalcoholic steatohepatitis: A systematic review and meta-analysis of paired-biopsy studies. Clin Gastroenterol Hepatol. 2015;13:643–54.e1–9. 24768810 10.1016/j.cgh.2014.04.014 PMC4208976

[28] Gidener T Ahmed OT Larson JJ Mara KC Therneau TM Venkatesh SK. Liver stiffness by magnetic resonance elastography predicts future cirrhosis, decompensation, and death in NAFLD. Clin Gastroenterol Hepatol. 2021;19:1915–1924.e6. 33010409 10.1016/j.cgh.2020.09.044 PMC9096913

[29] Han MAT Vipani A Noureddin N Ramirez K Gornbein J Saouaf R. Elastography-based liver fibrosis correlates with liver events in nonalcoholic fatty liver patients: A multicenter study. Liver Int. 2020;40:2242–2251. 32652744 10.1111/liv.14593

[30] Kim BK Bergstrom J Loomba R Tamaki N Izumi N Nakajima A. Magnetic resonance elastography-based prediction model for hepatic decompensation in NAFLD: A multicenter cohort study. Hepatology. 2023;78:1858–1866. 37203233 10.1097/HEP.0000000000000470 PMC10663382

[31] Lin H Lee HW Yip TC Tsochatzis E Petta S Bugianesi E.; VCTE-Prognosis Study Group. Vibration-controlled transient elastography scores to predict liver-related events in steatotic liver disease. JAMA. 2024;331:1287–1297. 38512249 10.1001/jama.2024.1447 PMC10958386

[32] Petta S Sebastiani G Viganò M Ampuero J Wai-Sun Wong V Boursier J. Monitoring occurrence of liver-related events and survival by transient elastography in patients with nonalcoholic fatty liver disease and compensated advanced chronic liver disease. Clin Gastroenterol Hepatol. 2021;19:806–815.e5. 32621970 10.1016/j.cgh.2020.06.045

[33] Lee HW Wong GL Kwok R Choi KC Chan CK Shu SS. Serial transient elastography examinations to monitor patients with type 2 diabetes: A prospective cohort study. Hepatology. 2020;72:1230–1241. 31991487 10.1002/hep.31142

[34] Tsochatzis EA Valenti L Thiele M Péloquin S Lazure P Masson MH. Use of non-invasive diagnostic tools for metabolic dysfunction-associated steatohepatitis: A qualitative exploration of challenges and barriers. Liver Int. 2024;44:1990–2001. 38634796 10.1111/liv.15941

[35] Younossi ZM Ong JP Takahashi H Yilmaz Y Eguc Hi Y El Kassas M. Global Nonalcoholic Steatohepatitis Council. A global survey of physicians knowledge about nonalcoholic fatty liver disease. Clin Gastroenterol Hepatol. 2022;20:e1456–e1468. 34229038 10.1016/j.cgh.2021.06.048

[36] Patel PJ Banh X Horsfall LU Hayward KL Hossain F Johnson T. Underappreciation of non-alcoholic fatty liver disease by primary care clinicians: Limited awareness of surrogate markers of fibrosis. Intern Med J. 2018;48:144–151. 29083080 10.1111/imj.13667

[37] Canivet CM Smati S Lannes A Brisseau J Judon L Roch ML. Awareness of chronic liver diseases, a comparison between diabetologists and general practitioners. Clin Res Hepatol Gastroenterol. 2022;46:101848. 34922062 10.1016/j.clinre.2021.101848

[38] Standing HC Jarvis H Orr J Exley C Hudson M Kaner E. GPs’ experiences and perceptions of early detection of liver disease: A qualitative study in primary care. Br J Gen Pract. 2018;68:e743–e749. 30249611 10.3399/bjgp18X699377 PMC6193778

[39] Eskridge W Cryer DR Schattenberg JM Gastaldelli A Malhi H Allen AM. Metabolic dysfunction–associated steatotic liver disease and metabolic dysfunction–associated steatohepatitis: The patient and physician perspective. J Clin Med. 2023;12:6216. 37834859 10.3390/jcm12196216 PMC10573476

[40] Noureddin M Charlton MR Harrison SA Bansal MB Alkhouri N Loomba R. Expert panel recommendations: Practical clinical applications for initiating and monitoring resmetirom in patients with MASH/NASH and moderate to noncirrhotic advanced fibrosis. Clin Gastroenterol Hepatol. 2024;22:2367–2377. 39038768 10.1016/j.cgh.2024.07.003

[41] Lin H Yip TC Zhang X Li G Tse YK Hui VW. Age and the relative importance of liver-related deaths in nonalcoholic fatty liver disease. Hepatology. 2023;77:573–584. 35790018 10.1002/hep.32633

