# Supplementary material for: Global patterns of utilization of noninvasive tests for the clinical management of metabolic dysfunction–associated steatotic liver disease
Source: Hepatol Commun. 2025 Apr 30;9(5):e0678. doi: 10.1097/HC9.0000000000000678 (PMC12045536; doi:10.1097/HC9.0000000000000678)
Supplement: Supplementary file 1 [file hc9-9-e0678-s001.docx]

**Supplementary Table 1.** Country of practice for the survey completers.

| Country | Hepatologists | Gastroenterologists | Others | All |
| --- | --- | --- | --- | --- |
| Algeria | 5 (2.9%) | 0 (0.0%) | 0 (0.0%) | 5 (1.6%) |
| Argentina | 0 (0.0%) | 0 (0.0%) | 1 (1.8%) | 1 (0.3%) |
| Australia | 2 (1.2%) | 0 (0.0%) | 0 (0.0%) | 2 (0.6%) |
| Bangladesh | 1 (0.6%) | 0 (0.0%) | 0 (0.0%) | 1 (0.3%) |
| Brazil | 5 (2.9%) | 4 (4.4%) | 0 (0.0%) | 9 (2.8%) |
| Cameroon | 1 (0.6%) | 0 (0.0%) | 0 (0.0%) | 1 (0.3%) |
| Canada | 1 (0.6%) | 0 (0.0%) | 2 (3.5%) | 3 (0.9%) |
| Chile | 0 (0.0%) | 2 (2.2%) | 0 (0.0%) | 2 (0.6%) |
| China | 12 (6.9%) | 10 (11.0%) | 1 (1.8%) | 23 (7.2%) |
| Cuba | 0 (0.0%) | 2 (2.2%) | 0 (0.0%) | 2 (0.6%) |
| Cyprus | 0 (0.0%) | 0 (0.0%) | 1 (1.8%) | 1 (0.3%) |
| Denmark | 4 (2.3%) | 0 (0.0%) | 0 (0.0%) | 4 (1.2%) |
| Egypt | 21 (12.1%) | 6 (6.6%) | 1 (1.8%) | 28 (8.7%) |
| Ethiopia | 0 (0.0%) | 2 (2.2%) | 0 (0.0%) | 2 (0.6%) |
| France | 5 (2.9%) | 0 (0.0%) | 6 (10.5%) | 11 (3.4%) |
| Germany | 0 (0.0%) | 1 (1.1%) | 2 (3.5%) | 3 (0.9%) |
| Ghana | 0 (0.0%) | 0 (0.0%) | 1 (1.8%) | 1 (0.3%) |
| Greece | 5 (2.9%) | 5 (5.5%) | 3 (5.3%) | 13 (4.0%) |
| India | 3 (1.7%) | 3 (3.3%) | 0 (0.0%) | 6 (1.9%) |
| Iran | 0 (0.0%) | 6 (6.6%) | 0 (0.0%) | 6 (1.9%) |
| Israel | 0 (0.0%) | 0 (0.0%) | 2 (3.5%) | 2 (0.6%) |
| Italy | 3 (1.7%) | 1 (1.1%) | 3 (5.3%) | 7 (2.2%) |
| Japan | 3 (1.7%) | 0 (0.0%) | 1 (1.8%) | 4 (1.2%) |
| Korea | 2 (1.2%) | 0 (0.0%) | 0 (0.0%) | 2 (0.6%) |
| Kuwait | 0 (0.0%) | 1 (1.1%) | 0 (0.0%) | 1 (0.3%) |
| Malaysia | 1 (0.6%) | 0 (0.0%) | 0 (0.0%) | 1 (0.3%) |
| Mexico | 2 (1.2%) | 0 (0.0%) | 0 (0.0%) | 2 (0.6%) |
| Moldova | 1 (0.6%) | 1 (1.1%) | 0 (0.0%) | 2 (0.6%) |
| Mongolia | 0 (0.0%) | 2 (2.2%) | 0 (0.0%) | 2 (0.6%) |
| Myanmar | 1 (0.6%) | 0 (0.0%) | 0 (0.0%) | 1 (0.3%) |
| Oman | 1 (0.6%) | 0 (0.0%) | 0 (0.0%) | 1 (0.3%) |
| Panama | 0 (0.0%) | 1 (1.1%) | 0 (0.0%) | 1 (0.3%) |
| Peru | 0 (0.0%) | 1 (1.1%) | 0 (0.0%) | 1 (0.3%) |
| Philippines | 1 (0.6%) | 0 (0.0%) | 0 (0.0%) | 1 (0.3%) |
| Portugal | 0 (0.0%) | 0 (0.0%) | 1 (1.8%) | 1 (0.3%) |
| Saudi Arabia | 8 (4.6%) | 7 (7.7%) | 0 (0.0%) | 15 (4.7%) |
| Spain | 9 (5.2%) | 2 (2.2%) | 17 (29.8%) | 28 (8.7%) |
| Sweden | 8 (4.6%) | 2 (2.2%) | 0 (0.0%) | 10 (3.1%) |
| Sudan | 0 (0.0%) | 1 (1.1%) | 0 (0.0%) | 1 (0.3%) |
| Taiwan | 8 (4.6%) | 0 (0.0%) | 1 (1.8%) | 9 (2.8%) |
| Thailand | 2 (1.2%) | 0 (0.0%) | 0 (0.0%) | 2 (0.6%) |
| Turkey | 3 (1.7%) | 12 (13.2%) | 6 (10.5%) | 21 (6.5%) |
| USA | 55 (31.8%) | 19 (20.9%) | 8 (14.0%) | 82 (25.5%) |

**Supplementary Table 2**. The use of NITs and respective cutoffs to determine the risk of significant (F2-F4) or advanced (F3-F4) fibrosis in patients with MASLD/NAFLD by the primary medical specialty of the survey completers.

| Question | Hepatologists | Gastroenterologists | Others | p |
| --- | --- | --- | --- | --- |
| **The most common NITs used to exclude (rule out) patients at risk of clinically significant fibrosis (F2-F4):** | | | | |
| ALT and AST | 22 (14.8%) | 18 (24.7%) | 15 (34.1%) | 0.0129 |
| FIB-4 | 108 (72.5%) | 55 (75.3%) | 38 (86.4%) | 0.17 |
| NFS | 13 (8.7%) | 9 (12.3%) | 10 (22.7%) | 0.0428 |
| FibroTest | 3 (2.0%) | 4 (5.5%) | 4 (9.1%) | 0.09 |
| ELF | 26 (17.4%) | 5 (6.8%) | 3 (6.8%) | 0.0365 |
| TE (FibroScan) | 138 (92.6%) | 66 (90.4%) | 34 (77.3%) | 0.0137 |
| FAST score | 12 (8.1%) | 5 (6.8%) | 1 (2.3%) | 0.41 |
| MRE | 34 (22.8%) | 9 (12.3%) | 0 (0.0%) | 0.0008 |
| 2D-SWE | 13 (8.7%) | 6 (8.2%) | 2 (4.5%) | 0.66 |
| Agile-3 | 3 (2.0%) | 1 (1.4%) | 1 (2.3%) | 0.93 |
| Agile-4 | 3 (2.0%) | 0 (0.0%) | 0 (0.0%) | 0.30 |
| **The order of NIT use and cutoffs to exclude (rule out) patients at risk of clinically significant fibrosis (F2-F4):** | | | | |
| FIB-4 is the first NIT of choice | 82 (55.0%) | 37 (50.7%) | 32 (72.7%) | 0.05 |
| FIB-4 is the second NIT of choice | 22 (14.8%) | 13 (17.8%) | 3 (6.8%) | 0.25 |
| (<65 years) FIB-4 < 1.00 | 3 (2.9%) | 3 (5.7%) | 0 (0.0%) | 0.31 |
| (<65 years) FIB-4 < 1.30 | 72 (70.6%) | 24 (45.3%) | 30 (81.1%) | 0.0006 |
| (<65 years) FIB-4 < 1.45 | 17 (16.7%) | 11 (20.8%) | 5 (13.5%) | 0.66 |
| (<65 years) FIB-4 < 2.00 | 2 (2.0%) | 5 (9.4%) | 1 (2.7%) | 0.08 |
| (<65 years) FIB-4 < 2.67 | 7 (6.9%) | 7 (13.2%) | 0 (0.0%) | 0.06 |
| (<65 years) FIB-4 < 3.25 | 1 (1.0%) | 1 (1.9%) | 1 (2.7%) | 0.75 |
| (<65 years) FIB-4 < other value | 0 (0.0%) | 2 (3.8%) | 0 (0.0%) | 0.07 |
| (≥65 years) FIB-4 < 1.00 | 2 (2.0%) | 0 (0.0%) | 1 (2.7%) | 0.53 |
| (≥65 years) FIB-4 < 1.30 | 21 (20.6%) | 7 (13.2%) | 10 (27.0%) | 0.26 |
| (≥65 years) FIB-4 < 1.45 | 21 (20.6%) | 17 (32.1%) | 5 (13.5%) | 0.09 |
| (≥65 years) FIB-4 < 2.00 | 45 (44.1%) | 17 (32.1%) | 17 (45.9%) | 0.28 |
| (≥65 years) FIB-4 < 2.67 | 12 (11.8%) | 7 (13.2%) | 3 (8.1%) | 0.75 |
| (≥65 years) FIB-4 < 3.25 | 1 (1.0%) | 4 (7.5%) | 1 (2.7%) | 0.08 |
| (≥65 years) FIB-4 < other value | 0 (0.0%) | 1 (1.9%) | 0 (0.0%) | 0.27 |
| NFS is the first NIT of choice | 4 (2.7%) | 1 (1.4%) | 3 (6.8%) | 0.23 |
| NFS is the second NIT of choice | 9 (6.0%) | 5 (6.8%) | 5 (11.4%) | 0.48 |
| NFS < -1.455 | 11 (68.8%) | 6 (66.7%) | 6 (66.7%) | 0.99 |
| NFS < 0.675 | 5 (31.3%) | 3 (33.3%) | 3 (33.3%) | 0.99 |
| ELF is the first NIT of choice | 3 (2.0%) | 1 (1.4%) | 0 (0.0%) | 0.62 |
| ELF is the second NIT of choice | 12 (8.1%) | 2 (2.7%) | 1 (2.3%) | 0.16 |
| ELF < 7.7 | 11 (42.3%) | 2 (40.0%) | 0 (0.0%) | 0.50 |
| ELF < 9.8 | 15 (57.7%) | 3 (60.0%) | 1 (50.0%) | 0.97 |
| ELF < 11.3 | 0 (0.0%) | 0 (0.0%) | 1 (50.0%) | 0.0003 |
| TE is the first NIT of choice | 54 (36.2%) | 21 (28.8%) | 5 (11.4%) | 0.0065 |
| TE is the second NIT of choice | 73 (49.0%) | 33 (45.2%) | 20 (45.5%) | 0.84 |
| TE < 6 kPa | 6 (4.4%) | 5 (7.9%) | 2 (5.9%) | 0.61 |
| TE < 7 kPa | 34 (25.2%) | 16 (25.4%) | 4 (11.8%) | 0.23 |
| TE < 8 kPa | 70 (51.9%) | 23 (36.5%) | 22 (64.7%) | 0.0213 |
| TE < 9 kPa | 13 (9.6%) | 7 (11.1%) | 3 (8.8%) | 0.92 |
| TE < 10 kPa | 7 (5.2%) | 7 (11.1%) | 3 (8.8%) | 0.31 |
| TE < 11 kPa | 1 (0.7%) | 1 (1.6%) | 0 (0.0%) | 0.70 |
| TE < 12 kPa | 3 (2.2%) | 3 (4.8%) | 0 (0.0%) | 0.34 |
| TE < other value | 1 (0.7%) | 1 (1.6%) | 0 (0.0%) | 0.70 |
| MRE is the first NIT of choice | 5 (3.4%) | 1 (1.4%) | 0 (0.0%) | 0.35 |
| MRE is the second NIT of choice | 5 (3.4%) | 3 (4.1%) | 0 (0.0%) | 0.42 |
| MRE < 3.14 kPa | 14 (41.2%) | 4 (44.4%) | 0 (0.0%) | 0.86 |
| MRE < 3.3 kPa | 10 (29.4%) | 3 (33.3%) | 0 (0.0%) | 0.82 |
| MRE < 3.5 kPa | 8 (23.5%) | 2 (22.2%) | 0 (0.0%) | 0.93 |
| MRE < 3.6 kPa | 2 (5.9%) | 0 (0.0%) | 0 (0.0%) | 0.46 |
| **The most common NITs used to exclude (rule out) patients at risk of advanced fibrosis (F3-F4):** | | | | |
| ALT and AST | 22 (15.1%) | 12 (16.9%) | 11 (26.2%) | 0.24 |
| FIB-4 | 97 (66.4%) | 40 (56.3%) | 31 (73.8%) | 0.14 |
| NFS | 15 (10.3%) | 7 (9.9%) | 8 (19.0%) | 0.25 |
| FibroTest | 5 (3.4%) | 3 (4.2%) | 2 (4.8%) | 0.91 |
| ELF | 25 (17.1%) | 3 (4.2%) | 2 (4.8%) | 0.0066 |
| TE (FibroScan) | 134 (91.8%) | 66 (93.0%) | 33 (78.6%) | 0.0263 |
| FAST score | 7 (4.8%) | 3 (4.2%) | 0 (0.0%) | 0.36 |
| MRE | 35 (24.0%) | 8 (11.3%) | 1 (2.4%) | 0.0015 |
| 2D-SWE | 12 (8.2%) | 6 (8.5%) | 2 (4.8%) | 0.73 |
| Agile-3 | 7 (4.8%) | 3 (4.2%) | 1 (2.4%) | 0.79 |
| Agile-4 | 3 (2.1%) | 2 (2.8%) | 0 (0.0%) | 0.57 |
| **The order of NIT use and cutoffs to exclude (rule out) patients at risk of advanced fibrosis (F3-F4):** | | | | |
| FIB-4 is the first NIT of choice | 73 (50.0%) | 28 (39.4%) | 25 (59.5%) | 0.11 |
| FIB-4 is the second NIT of choice | 19 (13.0%) | 9 (12.7%) | 5 (11.9%) | 0.98 |
| (<65 years) FIB-4 < 1.00 | 0 (0.0%) | 1 (2.6%) | 1 (3.2%) | 0.25 |
| (<65 years) FIB-4 < 1.30 | 39 (41.5%) | 16 (41.0%) | 14 (45.2%) | 0.93 |
| (<65 years) FIB-4 < 1.45 | 14 (14.9%) | 7 (17.9%) | 3 (9.7%) | 0.62 |
| (<65 years) FIB-4 < 2.00 | 5 (5.3%) | 3 (7.7%) | 1 (3.2%) | 0.71 |
| (<65 years) FIB-4 < 2.67 | 22 (23.4%) | 8 (20.5%) | 9 (29.0%) | 0.70 |
| (<65 years) FIB-4 < 3.25 | 14 (14.9%) | 3 (7.7%) | 3 (9.7%) | 0.46 |
| (<65 years) FIB-4 < other value | 0 (0.0%) | 1 (2.6%) | 0 (0.0%) | 0.20 |
| (≥65 years) FIB-4 < 1.00 | 0 (0.0%) | 1 (2.6%) | 0 (0.0%) | 0.20 |
| (≥65 years) FIB-4 < 1.30 | 10 (10.6%) | 3 (7.7%) | 6 (19.4%) | 0.29 |
| (≥65 years) FIB-4 < 1.45 | 18 (19.1%) | 14 (35.9%) | 3 (9.7%) | 0.0212 |
| (≥65 years) FIB-4 < 2.00 | 28 (29.8%) | 9 (23.1%) | 8 (25.8%) | 0.71 |
| (≥65 years) FIB-4 < 2.67 | 15 (16.0%) | 6 (15.4%) | 9 (29.0%) | 0.23 |
| (≥65 years) FIB-4 < 3.25 | 23 (24.5%) | 5 (12.8%) | 4 (12.9%) | 0.18 |
| (≥65 years) FIB-4 < other value | 0 (0.0%) | 1 (2.6%) | 1 (3.2%) | 0.25 |
| NFS is the first NIT of choice | 2 (1.4%) | 1 (1.4%) | 3 (7.1%) | 0.08 |
| NFS is the second NIT of choice | 9 (6.2%) | 4 (5.6%) | 4 (9.5%) | 0.69 |
| NFS < -1.455 | 5 (35.7%) | 4 (57.1%) | 5 (62.5%) | 0.42 |
| NFS < 0.675 | 9 (64.3%) | 3 (42.9%) | 3 (37.5%) | 0.42 |
| ELF is the first NIT of choice | 5 (3.4%) | 1 (1.4%) | 0 (0.0%) | 0.36 |
| ELF is the second NIT of choice | 10 (6.8%) | 1 (1.4%) | 1 (2.4%) | 0.15 |
| ELF < 7.7 | 3 (12.0%) | 1 (33.3%) | 0 (0.0%) | 0.55 |
| ELF < 9.8 | 18 (72.0%) | 1 (33.3%) | 1 (100.0%) | 0.31 |
| ELF < 11.3 | 4 (16.0%) | 1 (33.3%) | 0 (0.0%) | 0.68 |
| TE is the first NIT of choice | 61 (41.8%) | 30 (42.3%) | 12 (28.6%) | 0.27 |
| TE is the second NIT of choice | 62 (42.5%) | 27 (38.0%) | 17 (40.5%) | 0.82 |
| TE < 6 kPa | 2 (1.5%) | 2 (3.1%) | 3 (9.4%) | 0.07 |
| TE < 7 kPa | 13 (9.9%) | 9 (13.8%) | 0 (0.0%) | 0.09 |
| TE < 8 kPa | 39 (29.8%) | 9 (13.8%) | 14 (43.8%) | 0.0047 |
| TE < 9 kPa | 18 (13.7%) | 10 (15.4%) | 4 (12.5%) | 0.92 |
| TE < 10 kPa | 33 (25.2%) | 15 (23.1%) | 9 (28.1%) | 0.86 |
| TE < 11 kPa | 3 (2.3%) | 2 (3.1%) | 1 (3.1%) | 0.93 |
| TE < 12 kPa | 21 (16.0%) | 16 (24.6%) | 1 (3.1%) | 0.0271 |
| TE < other value | 2 (1.5%) | 2 (3.1%) | 0 (0.0%) | 0.53 |
| MRE is the first NIT of choice | 4 (2.7%) | 1 (1.4%) | 0 (0.0%) | 0.49 |
| MRE is the second NIT of choice | 8 (5.5%) | 3 (4.2%) | 0 (0.0%) | 0.30 |
| MRE < 3.14 kPa | 6 (17.1%) | 1 (12.5%) | 0 (0.0%) | 0.86 |
| MRE < 3.3 kPa | 7 (20.0%) | 1 (12.5%) | 0 (0.0%) | 0.79 |
| MRE < 3.5 kPa | 8 (22.9%) | 5 (62.5%) | 1 (100.0%) | 0.0316 |
| MRE < 3.6 kPa | 10 (28.6%) | 1 (12.5%) | 0 (0.0%) | 0.54 |
| MRE < other kPa | 4 (11.4%) | 0 (0.0%) | 0 (0.0%) | 0.57 |

**Supplementary Table 3**. Professional society risk-stratification pathways used by the survey completers.

| Question | Hepatologists | Gastroenterologists | Others | p | All |
| --- | --- | --- | --- | --- | --- |
| Do you use a formal/written professional society risk stratification pathway?  N (%) “Yes” | 114 (72.2%) | 37 (49.3%) | 32 (65.3%) | 0.0030 | 183 (64.9%) |
| Which society? (free text) |  |  |  |  |  |
| AACE | 5 (5.4%) | 0 (0.0%) | 6 (25.0%) | 0.0013 | 11 (7.6%) |
| AASLD | 46 (50.0%) | 14 (48.3%) | 2 (8.3%) | 0.0009 | 62 (42.8%) |
| ACG | 1 (1.1%) | 0 (0.0%) | 0 (0.0%) | 0.75 | 1 (0.7%) |
| AGA | 11 (12.0%) | 2 (6.9%) | 2 (8.3%) | 0.69 | 15 (10.3%) |
| EASL | 19 (20.7%) | 4 (13.8%) | 9 (37.5%) | 0.10 | 32 (22.1%) |
| APASL | 1 (1.1%) | 0 (0.0%) | 0 (0.0%) | 0.75 | 1 (0.7%) |
| other | 25 (27.2%) | 12 (41.4%) | 6 (25.0%) | 0.30 | 43 (29.7%) |

**Supplementary Table 4.** Demographic parameters of the survey completers by the global region.

| Question | Europe | Middle East and Africa | Latin America | Asia | USA | p | All |
| --- | --- | --- | --- | --- | --- | --- | --- |
| N | 82 | 83 | 18 | 53 | 85 |  | 321 |
| What is your age group? |  |  |  |  |  |  |  |
| 25-34 years | 10 (12.2%) | 9 (10.8%) | 2 (11.1%) | 7 (13.2%) | 4 (4.7%) | 0.43 | 32 (10.0%) |
| 35-44 years | 21 (25.6%) | 37 (44.6%) | 2 (11.1%) | 13 (24.5%) | 22 (25.9%) | 0.0082 | 95 (29.6%) |
| 45-54 years | 18 (22.0%) | 29 (34.9%) | 7 (38.9%) | 14 (26.4%) | 21 (24.7%) | 0.28 | 89 (27.7%) |
| 55-70 years | 30 (36.6%) | 8 (9.6%) | 7 (38.9%) | 17 (32.1%) | 27 (31.8%) | 0.0008 | 89 (27.7%) |
| Above 70 years | 3 (3.7%) | 0 (0.0%) | 0 (0.0%) | 2 (3.8%) | 11 (12.9%) | 0.0018 | 16 (5.0%) |
| Male gender | 48 (58.5%) | 49 (59.8%) | 7 (38.9%) | 38 (71.7%) | 52 (61.2%) | 0.17 | 194 (60.6%) |
| Primary specialization/discipline: |  |  |  |  |  |  |  |
| Primary Care or General Medicine | 6 (7.3%) | 5 (6.0%) | 0 (0.0%) | 1 (1.9%) | 7 (8.2%) | 0.44 | 19 (5.9%) |
| Gastroenterology | 12 (14.6%) | 35 (42.2%) | 10 (55.6%) | 15 (28.3%) | 19 (22.4%) | 0.0001 | 91 (28.3%) |
| Hepatology | 37 (45.1%) | 38 (45.8%) | 7 (38.9%) | 35 (66.0%) | 56 (65.9%) | 0.0060 | 173 (53.9%) |
| Endocrinology | 23 (28.0%) | 1 (1.2%) | 0 (0.0%) | 1 (1.9%) | 1 (1.2%) | <.0001 | 26 (8.1%) |
| Medical weight loss | 2 (2.4%) | 2 (2.4%) | 0 (0.0%) | 0 (0.0%) | 0 (0.0%) | 0.43 | 4 (1.2%) |
| Other | 2 (2.4%) | 2 (2.4%) | 1 (5.6%) | 1 (1.9%) | 2 (2.4%) | 0.94 | 8 (2.5%) |
| Secondary specialization/discipline: |  |  |  |  |  |  |  |
| Primary Care or General Medicine | 5 (6.1%) | 3 (3.6%) | 1 (5.6%) | 2 (3.8%) | 6 (7.1%) | 0.85 | 17 (5.3%) |
| Gastroenterology | 17 (20.7%) | 40 (48.2%) | 7 (38.9%) | 24 (45.3%) | 26 (30.6%) | 0.0021 | 114 (35.5%) |
| Hepatology | 15 (18.3%) | 22 (26.5%) | 10 (55.6%) | 16 (30.2%) | 18 (21.2%) | 0.0148 | 81 (25.2%) |
| Endocrinology | 1 (1.2%) | 0 (0.0%) | 0 (0.0%) | 2 (3.8%) | 1 (1.2%) | 0.40 | 4 (1.2%) |
| Medical weight loss | 4 (4.9%) | 0 (0.0%) | 0 (0.0%) | 1 (1.9%) | 4 (4.7%) | 0.24 | 9 (2.8%) |
| Other | 10 (12.2%) | 5 (6.0%) | 0 (0.0%) | 1 (1.9%) | 7 (8.2%) | 0.13 | 23 (7.2%) |
| None | 30 (36.6%) | 13 (15.7%) | 0 (0.0%) | 7 (13.2%) | 23 (27.1%) | 0.0004 | 73 (22.7%) |
| Number of years in practice? |  |  |  |  |  |  |  |
| <5 years | 7 (8.5%) | 13 (15.7%) | 1 (5.6%) | 2 (3.8%) | 9 (10.6%) | 0.21 | 32 (10.0%) |
| 5-10 years | 9 (11.0%) | 11 (13.3%) | 3 (16.7%) | 6 (11.3%) | 12 (14.1%) | 0.95 | 41 (12.8%) |
| 11-20 years | 25 (30.5%) | 33 (39.8%) | 3 (16.7%) | 20 (37.7%) | 26 (30.6%) | 0.30 | 107 (33.3%) |
| >20 years | 41 (50.0%) | 26 (31.3%) | 11 (61.1%) | 25 (47.2%) | 38 (44.7%) | 0.06 | 141 (43.9%) |
| Clinic setting: |  |  |  |  |  |  |  |
| Academic | 47 (57.3%) | 30 (36.1%) | 8 (44.4%) | 27 (50.9%) | 48 (56.5%) | 0.0441 | 160 (49.8%) |
| Hospital | 33 (40.2%) | 39 (47.0%) | 7 (38.9%) | 20 (37.7%) | 14 (16.5%) | 0.0007 | 113 (35.2%) |
| Private practice | 2 (2.4%) | 14 (16.9%) | 3 (16.7%) | 5 (9.4%) | 20 (23.5%) | 0.0016 | 44 (13.7%) |
| Other | 0 (0.0%) | 0 (0.0%) | 0 (0.0%) | 1 (1.9%) | 3 (3.5%) | 0.19 | 4 (1.2%) |
| Medical degree: |  |  |  |  |  |  |  |
| MD | 80 (97.6%) | 75 (90.4%) | 18 (100.0%) | 51 (96.2%) | 65 (76.5%) | <.0001 | 289 (90.0%) |
| DO | 0 (0.0%) | 0 (0.0%) | 0 (0.0%) | 1 (1.9%) | 1 (1.2%) | 0.57 | 2 (0.6%) |
| NP or PA | 1 (1.2%) | 2 (2.4%) | 0 (0.0%) | 0 (0.0%) | 18 (21.2%) | <.0001 | 21 (6.5%) |
| Dietitian or Nutritionist | 0 (0.0%) | 2 (2.4%) | 0 (0.0%) | 0 (0.0%) | 0 (0.0%) | 0.22 | 2 (0.6%) |
| Exercise Specialist | 0 (0.0%) | 2 (2.4%) | 0 (0.0%) | 0 (0.0%) | 0 (0.0%) | 0.22 | 2 (0.6%) |
| Other | 1 (1.2%) | 2 (2.4%) | 0 (0.0%) | 1 (1.9%) | 1 (1.2%) | 0.93 | 5 (1.6%) |
| Region of practice: |  |  |  |  |  |  |  |
| a large city or urban area | 67 (81.7%) | 67 (80.7%) | 18 (100.0%) | 47 (88.7%) | 48 (56.5%) | <.0001 | 247 (76.9%) |
| a medium-sized city or suburb | 14 (17.1%) | 12 (14.5%) | 0 (0.0%) | 4 (7.5%) | 34 (40.0%) | <.0001 | 64 (19.9%) |
| a small town or rural area | 1 (1.2%) | 4 (4.8%) | 0 (0.0%) | 2 (3.8%) | 3 (3.5%) | 0.65 | 10 (3.1%) |
| MASLD patients seen per month (new or follow-up): |  |  |  |  |  |  |  |
| None | 1 (1.2%) | 1 (1.2%) | 0 (0.0%) | 0 (0.0%) | 1 (1.2%) | 0.93 | 3 (0.9%) |
| 1 to 19 | 25 (30.5%) | 33 (39.8%) | 2 (11.1%) | 6 (11.3%) | 22 (25.9%) | 0.0028 | 88 (27.4%) |
| 20 to 49 | 31 (37.8%) | 21 (25.3%) | 9 (50.0%) | 15 (28.3%) | 32 (37.6%) | 0.15 | 108 (33.6%) |
| > 50 | 25 (30.5%) | 28 (33.7%) | 7 (38.9%) | 32 (60.4%) | 30 (35.3%) | 0.0069 | 122 (38.0%) |
| Patients with MASLD/NAFLD or MASH/NASH are referred from: |  |  |  |  |  |  |  |
| Primary care | 54 (65.9%) | 60 (72.3%) | 7 (38.9%) | 38 (71.7%) | 78 (91.8%) | <.0001 | 237 (73.8%) |
| Other specialty such as Endocrinology | 41 (50.0%) | 45 (54.2%) | 10 (55.6%) | 36 (67.9%) | 51 (60.0%) | 0.31 | 183 (57.0%) |
| Own practice | 42 (51.2%) | 57 (68.7%) | 8 (44.4%) | 39 (73.6%) | 50 (58.8%) | 0.0251 | 196 (61.1%) |
| Select which NITs are available to exclude (rule out) patients at risk of significant or advanced fibrosis in your clinical practice: (Please choose all that apply) | | | | | | | |
| ALT and AST | 39 (47.6%) | 52 (68.4%) | 9 (52.9%) | 31 (62.0%) | 38 (48.7%) | 0.0480 | 169 (55.8%) |
| FIB-4 | 77 (93.9%) | 58 (76.3%) | 17 (100.0%) | 40 (80.0%) | 68 (87.2%) | 0.0063 | 260 (85.8%) |
| NFS | 27 (32.9%) | 13 (17.1%) | 6 (35.3%) | 15 (30.0%) | 13 (16.7%) | 0.0423 | 74 (24.4%) |
| FibroTest | 8 (9.8%) | 7 (9.2%) | 2 (11.8%) | 4 (8.0%) | 20 (25.6%) | 0.0097 | 41 (13.5%) |
| ELF | 7 (8.5%) | 1 (1.3%) | 1 (5.9%) | 4 (8.0%) | 28 (35.9%) | <.0001 | 41 (13.5%) |
| TE (FibroScan) | 77 (93.9%) | 65 (85.5%) | 16 (94.1%) | 41 (82.0%) | 70 (89.7%) | 0.21 | 269 (88.8%) |
| FAST score | 6 (7.3%) | 8 (10.5%) | 5 (29.4%) | 14 (28.0%) | 14 (17.9%) | 0.0060 | 47 (15.5%) |
| MRE | 14 (17.1%) | 7 (9.2%) | 6 (35.3%) | 13 (26.0%) | 44 (56.4%) | <.0001 | 84 (27.7%) |
| 2D-SWE | 16 (19.5%) | 3 (3.9%) | 10 (58.8%) | 15 (30.0%) | 7 (9.0%) | <.0001 | 51 (16.8%) |
| Agile-3 | 6 (7.3%) | 2 (2.6%) | 1 (5.9%) | 9 (18.0%) | 7 (9.0%) | 0.0454 | 25 (8.3%) |
| Agile-4 | 4 (4.9%) | 2 (2.6%) | 1 (5.9%) | 9 (18.0%) | 7 (9.0%) | 0.0212 | 23 (7.6%) |
| If your first-line NIT is FIB-4 and it is in the indeterminate range, you: |  |  |  |  |  |  |  |
| Perform a liver biopsy | 5 (7.7%) | 9 (16.4%) | 1 (6.3%) | 15 (34.9%) | 10 (14.7%) | 0.0033 | 40 (16.2%) |
| Perform another NIT | 50 (76.9%) | 36 (65.5%) | 14 (87.5%) | 22 (51.2%) | 52 (76.5%) | 0.0109 | 174 (70.4%) |
| Refer to a specialist (GI/hepatologist) | 10 (15.4%) | 6 (10.9%) | 1 (6.3%) | 3 (7.0%) | 5 (7.4%) | 0.51 | 25 (10.1%) |
| Nothing | 0 (0.0%) | 3 (5.5%) | 0 (0.0%) | 3 (7.0%) | 1 (1.5%) | 0.14 | 7 (2.8%) |
| Do not know | 0 (0.0%) | 1 (1.8%) | 0 (0.0%) | 0 (0.0%) | 0 (0.0%) | 0.48 | 1 (0.4%) |
| Do you use a formal/written professional society risk stratification pathway? N (%) “Yes” | 57 (73.1%) | 36 (51.4%) | 11 (73.3%) | 30 (62.5%) | 49 (69.0%) | 0.06 | 183 (64.9%) |
| Which society? (free text) |  |  |  |  |  |  |  |
| AACE | 4 (8.7%) | 0 (0.0%) | 0 (0.0%) | 0 (0.0%) | 7 (16.3%) | 0.05 | 11 (7.6%) |
| AASLD | 3 (6.5%) | 11 (47.8%) | 8 (80.0%) | 8 (34.8%) | 32 (74.4%) | <.0001 | 62 (42.8%) |
| ACG | 0 (0.0%) | 0 (0.0%) | 0 (0.0%) | 0 (0.0%) | 1 (2.3%) | 0.66 | 1 (0.7%) |
| AGA | 1 (2.2%) | 2 (8.7%) | 0 (0.0%) | 2 (8.7%) | 10 (23.3%) | 0.0151 | 15 (10.3%) |
| EASL | 19 (41.3%) | 6 (26.1%) | 4 (40.0%) | 3 (13.0%) | 0 (0.0%) | <.0001 | 32 (22.1%) |
| APASL | 0 (0.0%) | 0 (0.0%) | 0 (0.0%) | 1 (4.3%) | 0 (0.0%) | 0.25 | 1 (0.7%) |
| other | 22 (47.8%) | 6 (26.1%) | 2 (20.0%) | 12 (52.2%) | 1 (2.3%) | <.0001 | 43 (29.7%) |

**Supplementary material: The survey**

**Part 1**: Basic information

| **Question** | **Answer options** |
| --- | --- |
| What is your age group? | - 18-24 years - 25-34 years - 35-44 years - 45-54 years - 55-70 years - Above 70 years |
| Which of the following describes your gender? | - Man - Woman - Other - I prefer not to say |
| What is your primary area of specialization? (Value Required) | - Primary Care or General Medicine - Gastroenterology - Hepatology - Endocrinology - Medical weight loss specialty clinic - Other |
| Do you have any other specialization? (Value Required) | - Primary Care or General Medicine - Gastroenterology - Hepatology - Endocrinology - Medical weight loss specialty clinic - Other - None |
| Number of years in practice? | - <5 years - 5-10 years - 11-20 years - >20 years |
| Clinic setting | - Academic - Private practice - Hospital-associated - Other |
| What is your Professional Degree ? (Value Required) | - MD - DO - Advanced practice provider (NP or PA) - Nurse - Dietitian / Nutritionist - Exercise specialist - Psychologist / psychiatrist / behavioral health - Diabetes educator - Social worker - Other |
| What percentage of your work time is spent in clinical practice: (Value Required) | - 0-10% - 11-20% - 21-30% - 31-40% - 41-50% - 51-60% - 61-70% - 71-80% - 81-90% - 91-100% |
| Indicate the type of region in which you practice in: | - a large city or urban area - a medium-sized city or suburb - a small town or rural area |
| Country of Practice | Drop down to select country |
| In the past 12 months, how many patients on average with non-alcoholic fatty liver disease (NAFLD)/(MASLD) (new or follow up) do you see per month? | - None - 1-19 - 20-49 - >50 |
| How are MASLD/NAFLD/NASH patients identified or referred to your practice? (Please choose all that apply) | - From primary care - Other specialty such as Endocrinology - Own practice |
| Does your electronic medical record (EMR) automatically calculate FIB-4? | - Yes - No - I don’t know |

**Part 2.** Risk factors for advanced fibrosis.

**Which risk factors do you consider when deciding to assess patients for advanced fibrosis in known or suspected MASLD/NAFLD (multiple choice):**

- Age
- Diabetes mellitus type 1
- Diabetes mellitus type 2
- Prediabetes
- Insulin resistance
- Overweight
- Obesity
- Hypertension
- Dyslipidemia
- Elevated waist circumference (>35 inches female > 40 inches male)
- PCOS
- Abnormal liver enzymes
- Steatosis on imaging
- Obstructive sleep apnea
- Gout
- Use of steatogenic drugs
- Alcohol intake
- Everyone with suspected MASLD/NAFLD irrespective of risk factors

**Part 3**: NITs available and cut-offs employed to determine the risk of fibrosis in patients with MASLD/NAFLD in your clinic / department

**Please select which NITs are available to exclude (rule out) patients at risk of significant or advanced fibrosis in your clinical practice:** (Please choose all that apply)

- AST/ALT
- FIB-4
- NFS
- FibroTest
- ELF
- TE (FibroScan)
- FAST score
- MRE
- 2D-SWE
- Agile 3+
- Agile 4

**3.1 Do you use NITs to exclude patients at risk of clinically significant (F2-F4) fibrosis in your clinical practice?**

- Yes
- No

*If “No”, the survey proceeds to Part 3.2.*

**Please select the most common NITs (please choose up to 3 that apply) you use to exclude (rule out) patients at risk of clinically significant fibrosis (F2-F4) in your clinical practice:**

- AST/ALT
- FIB-4
- NFS
- FibroTest
- ELF
- TE (FibroScan)
- FAST score
- MRE
- 2D-SWE
- Agile 3+
- Agile 4

**Please select the most common NITs (please choose up to 3 that apply) you use to exclude (rule out) patients at risk of advanced fibrosis (F3-F4) in your clinical practice:**

- AST/ALT
- FIB-4
- NFS
- FibroTest
- ELF
- TE (FibroScan)
- FAST score
- MRE
- 2D-SWE
- Agile 3+
- Agile 4

*For any chosen NIT, there will be a list of options to choose from, for F2-F4 and F3-F4 choices separately. If an NIT is not selected, the respective options will not be shown.*

*Each NIT chosen above has to be indicated as of in which order it would be used*

**Please specify <NIT> use order**

- 1^st^
- 2^nd^
- …
- 9^th^

*Each NIT chosen above comes with a list of cutoff options to choose from (a respective dropdown menu is shown):*

- FIB-4 cutoff
  - - For age <65: <1.0, 1.3, 1.45, 2.0, 2.67, 3.25, other
    - For age ≥65: <1.0, 1.3, 1.45, 2.0, 2.67, 3.25, other
- NFS cutoff
  - - <-1.455
    - 0.675
    - other
- FibroTest cutoff
- <0.21
- <0.27
- <0.31
- <0.48
  - - other
- ELF cutoff
  - - <7.7
    - <9.8
    - <11.3
    - other
- TE (Fibroscan) cutoff
  - - LSM<6 kPa
    - LSM<7 kPa
    - LSM<8 kPa
    - LSM<9 kPa
    - LSM<10 kPa
    - LSM<11 kPa
    - LSM<12 kPa
    - other
- FAST score cutoff
  - - < 0.35
    - <0.6
    - other
- MRE cutoff
  - - LSM<3.14kPa
    - LSM<3.3 kPa
    - LSM<3.5 kPa
    - LSM<3.6 kPa
    - other
- 2D-SWE
  - - <5 kPa
    - <9 kPa
    - <13 kPa
    - <17 kPa
    - other
- Agile 3+ cutoff
  - - <0.451
    - <0.678
    - other
- Agile 4 cutoff
  - - <0.251
    - <0.842
    - other

*If “other” is chosen, a free text form with “<NIT> other cutoff details” would be shown.*

**3.2 Do you use NITs to identify patients at risk of advanced fibrosis (F3-F4) in your clinical practice?**

- Yes
- No

*If “No”, the survey proceeds to Part 4.*

**Please select the most common NITs you use to identify (rule in) patients at risk for advanced fibrosis (F3-F4) and the cut off value in your clinical practice and the corresponding action.**

**First NIT you use**

- AST/ALT
- FIB-4
- NFS
- FibroTest
- ELF
- TE (FibroScan)
- FAST score
- MRE
- 2D-SWE
- Agile 3+
- Agile 4

*For each checked NIT, a dropdown menu for cutoffs (***Cutoff***) and action options (***Diagnosis type***) is shown.*

*The action options are identical for all NITs as follows:*

**Diagnosis type**

- Sufficient to make a definitive diagnosis of advanced fibrosis
- Suspect advanced fibrosis and …

**Next step***:*

- - Refer to specialty care
  - Confirm with another NIT
    - 2^nd^ NIT: drop down to choices
    - 3^rd^ NIT: drop down to choices
    - Confirm with liver biopsy
    - Do nothing
    - I don’t know

*The cutoff options are NIT-specific as follows:*

**Cutoff**

FIB-4 > : 1.3, 1.45, 2, 2.67, 3.25

NFS > : -1.455, 0.675

FibroTest > : 0.49, 0.59, 0.73

ELF > : 7.7, 9.8, 11.3

TE (Fibroscan) LSM > : 8, 9, 10, 12, 15, 20 kPa

FAST score > : 0.67

MRE LSM > : 3.14, 3.30, 3.53, 3.60 kPa

2D-SWE > : 5, 9, 13, 17 kPa

Agile 3+ > : 0.451, 0.678

Agile 4 > : 0.251, 0.842

**If your first-line NIT is in the indeterminate range you ...**

- Perform a liver biopsy
- Perform another NIT
- Refer for specialty evaluation by GI/Hepatology (For Primary care providers or Endocrinologist)
- Do nothing
- I don’t know

**Do you use different NITs or different cut-off for people with diabetes?**

- No
- Yes: please specify first line NIT and cutoff *(the choice of NITs and NIT-specific cutoff options are the same as above)*
- N/A - I only see people with diabetes

**What proportion of your MASLD/NAFLD patients do you biopsy or refer for liver biopsy outside of clinical trials?**

- 0-10%
- 11-20%
- 21-30%
- 31-40%
- 41-50%
- 51-60%
- 61-70%
- 71-80%
- 81-90%
- 91-100%

**Part 4**. NITs used and cut-offs employed to monitor disease progression or treatment response in MASLD/NAFLD patients with advanced fibrosis in your clinic / department

**Do you monitor disease progression or response in MASLD/NAFLD patients with low risk of advanced fibrosis (e.g. LSM<8 kPa by transient elastography or FIB-4 < 1.3)?**

- Yes
- No

*If Yes:*

**Which test:**

- FIB-4
- NFS
- FibroTest
- ELF
- TE (FibroScan)
- FAST score
- MRE
- 2D-SWE
- Agile 3+
- Agile 4
- Liver biopsy

**At what interval:**

- Every year
- Every 2 years
- Every 3 years
- Every 4 years
- Every 5 years

**Do you monitor disease progression or response in MASLD/NAFLD patients with intermediate risk of advanced fibrosis?**

- Yes
- No

*If Yes:*

**Which test:**

- FIB-4
- NFS
- FibroTest
- ELF
- TE (FibroScan)
- FAST score
- MRE
- 2D-SWE
- Agile 3+
- Agile 4
- Liver biopsy

**At what interval:**

- Every year
- Every 2 years
- Every 3 years
- Every 4 years
- Every 5 years

**Do you monitor disease progression or response in MASLD/NAFLD patients with high risk of advanced fibrosis?**

- Yes
- No

*If Yes:*

**Which test:**

- FIB-4
- NFS
- FibroTest
- ELF
- TE (FibroScan)
- FAST score
- MRE
- 2D-SWE
- Agile 3+
- Agile 4
- Liver biopsy

**At what interval:**

- Every year
- Every 2 years
- Every 3 years
- Every 4 years
- Every 5 years

**Part 5.** Formal care pathways and supporting documentation.

**Do you use a formal / written national / professional society (e.g. AASLD) risk stratification pathway ?**

- Yes
- No
- Not aware

*If Yes*

**Does this national / professional pathway outline the NITs and the cut-offs ?**

- Yes
- No

**Please provide the name of the national / professional pathway (if available)**

*<Free text form>*

**Do you use a formal / written local / regional risk stratification pathway ?**

- Yes
- No
- Not aware

*If Yes*

**Does this local / regional pathway outline the NITs and the cut-offs ?**

- Yes
- No

**Please provide the name of the local / regional pathway (if available)**

*<Free text form>*

**Please put any comments or feedback in the box below**
